# The predictive validity and effects of using the transtheoretical model to increase the physical activity of healthcare workers in a public hospital in South Africa

**DOI:** 10.1007/s13142-012-0136-5

**Published:** 2012-05-31

**Authors:** Linda Skaal, Supa Pengpid

**Affiliations:** 1 The Department of Social and Behavioural Health Sciences, School of Public Health, University of Limpopo (MEDUNSA Campus), Pretoria, South Africa; 2 The Department of Health System Management and Policy, School of Public Health, University of Limpopo (MEDUNSA Campus), Pretoria, South Africa; 3 P.O. Box 1018, Medunsa, Garankuwa 0204, Akasia, Pretoria, South Africa

**Keywords:** Transtheoretical model, Behavior change, Healthcare workers, Physical activity

## Abstract

There have been studies conducted on the effectiveness of the transtheoretical model (TTM) in improving the level of physical activity at worksites worldwide, but no such studies have been conducted in South Africa. The aim of this study was to determine the predictive validity and effects of using the Transtheoretical Model to increase the physical activity of healthcare workers in a public hospital in South Africa. A quasi-experimental design in the form of a single-group, pretest–posttest model was used to examine the possible relationship between an exposure to interventions, attitude, knowledge, and an increased level of physical activity. Two hundred hospital staff members (medical and nonmedical staff) were randomly selected for participation in the study. The following variables were measured: TTM stages of physical activity, knowledge and attitudes, fitness level, body mass index, and level of exposure to the intervention. The interventions designed were based on the concept of progressing stages of physical activity in TTM stage sequences: (1) pamphlets about physical activity and health, (2) posters, fun runs, and sports day, and (3) a second set of posters, a daily radio program, and aerobic classes. Post-intervention, participants had significantly increased their stages of physical activity, attitudes, and knowledge compared with their pre-tests. Mean scores of TTM (3.70) and knowledge (3.65) were significantly (*p* < 0.05) greater at post-test. Overall accuracies of TTM at pre-test correctly predicted TTM at post-test by an average of 66.9%. The use of TTM to identify the stage of physical activity of healthcare workers has enabled the researcher to design intervention programs specific to the stage of exercise behavior of hospital staff. The predictors (TTM1), exposure levels, knowledge, attitudes, and processes of change have significant contributions to the outcome (TTM2).

## BACKGROUND/INTRODUCTION

South Africa has a well-documented burden of obesity and chronic illnesses such as diabetes, hypertension, and cardiovascular disease. In 2002, a study found that 29.2% of black men and 56% of black women over the age of 35 years were overweight or obese in South Africa [1], and this figure has increased over the years. Because obesity is a risk factor for non-communicable diseases (NCDs), its high prevalence in South Africa has significantly contributed to the burden of NCDs, especially among blacks. By 2006, NCDs had accounted for 37% of deaths in the country; cardiovascular disease and diabetes together accounted for 19% of deaths [2]. Globally, physical inactivity (PI) has been estimated to cause approximately 10–16% of diabetes cases and approximately 22% of ischemic heart disease cases in both men and women. The World Health Organization (WHO) estimates the level of PI to be 17% among adults, despite the documented benefits of physical activity (PA) [3]. Even within a population that is physically active, it is reported that at least 60% of people fail to achieve the minimum recommendation of 30 min of moderate-intensity PA daily [4].

Healthcare workers are also exposed to these risk factors and are likely to suffer from NCDs. González-Velázquez and Mendez found that, in Mexico, 52% of female HCWs were obese, compared with only 23% of males [5]. Prevalences of hypertension, type 2 diabetes, hypercholesterolemia, and hypertriglyceridemia were 22%, 8%, 70%, and 47%, respectively [5]. These results demonstrate that HCWs are also at high risk of developing NCDs despite their professional status.

Research on the health of HCWs is minimally documented in South Africa; most studies concentrate on the management of patients in hospitals instead of the health of HCWs. As the majority of employees in hospitals are health professionals, it is assumed that they are aware of the health risks associated with PI and the benefits of participating in PA; however, there is no evidence to support this assumption.

The world is shifting towards the use of theory-based interventions to increase the level of PA. Glanz and Bishop (p. 399) stated that, “*Increasing evidence suggests that public health and health-promotion interventions that are based on social and behavioral science theories are more effective than those lacking a theoretical base.”* While little success in changing behavior is documented where no theory was used, theory-based interventions have had significant success in designing effective interventions that are guided by constructs of each theory to change people’s behaviors. Theory-based interventions have been associated with larger and longer-term effects than those without an explicit basis in theory [6]. A systemic review of 22 studies reporting on interventions with 5–11-year-old obese children to promote PA, of which most were not theory-based, revealed that only four interventions were successful in increasing the level of PA among the participants [7]. A challenge still exists on how to encourage individuals to initiate exercising, irrespective of their perceived barriers, age, gender, or cultural background.

South Africa has a diverse population with different cultural backgrounds, leading to differences in lifestyle including food consumption patterns and levels of PA. Furthermore, there are differences between rural and urban areas in terms of poverty, nutrition, and PA status, resulting in a high prevalence of obesity and NCDs in urban compared with rural areas [8]. The South African Department of Health has developed national guidelines that incorporate PA and are targeting older adults. Furthermore, there are several national campaigns aimed at increasing awareness regarding PA and health, such as the National Wellness Day. However, “these initiatives lack a broad-based infrastructure for implementation, as well as financial support and community awareness for sustainability,” according to Sparling et al. [9]. In addition, theory-based interventions are rarely used in this country, resulting in the failures of many of the existing interventions to have a lasting impact.

The transtheoretical model (TTM) of the stages and processes of change has been used to understand how people change problem behaviors such as PI [10]. The model suggests that individuals engaging in a new behavior move through a series of stages of change: pre-contemplation, contemplation, preparation, action, and maintenance [11]. Pre-contemplation is when an individual does not intend to change his/her high-risk behaviors in the foreseeable future. This stage would refer to a sedentary person with a very negative attitude and unwillingness to participate in PA. Contemplation is when an individual is giving serious consideration to behavior change—a person who is considering the need for an increased level of PA but is not yet exercising. Preparation refers to when an individual has made a decision to take action towards change in the near future—a sedentary person who has begun to make small changes in behavior and has a plan of action for change. Action is when an individual is actively participating in the new behavior and the behavior needs increase in expenditure of effort. Maintenance is the stage in which the new behavior has been demonstrated for an extended period of time and the new behavior requires less effort to maintain.

The TTM utilizes the concepts of stages of change and processes of change in a format that allows for the design of relevant interventions to facilitate behavior change for target populations [10]. This model can be used to predict addictive behavior and exercise adherence and categorizes people into different stages with different processes used to move people to the next stage. It is therefore important to identify the individual’s current stage and then use stage-specific strategies known to be effective for each stage. There is sufficient evidence that this model has been successfully used to guide exercise behaviors in Europe and America and has demonstrated a high predictive power for self-reported PA in numerous studies [12–14]. The aim of this study was to determine the predictive validity and effects of using the TTM to increase PA among HCWs in a public hospital in South Africa.

## METHODS

### Research design

A quasi-experimental design in the form of single-group, pretest–posttest model was used to examine the possible relationship between an exposure to interventions and changes in attitudes, knowledge, and levels of PA.

### Participants

The sample size was calculated based on 80% power and a 5% significance level with 25% at regular physical activity before the intervention and 50% at regular physical activity for post-intervention. The minimum sample size required was 58 individuals per group. To account for attrition of participants during the study, the sample size was increased to 200 (100 medical and 100 nonmedical staff). The participants were randomly selected from a list of staff members obtained from the human resources department. At post-test, only 163 staff (83 medical and 80 nonmedical staff) participated. The attrition rate was 18.5%. However, the remaining sample was greater than the minimum sample size calculated. An ethical clearance certificate (No. MREC/PH/82/2008: PG) and trial registration number were obtained from the University of Limpopo, and permission to conduct the study was provided by Hospital Management. Participants gave their informed consent prior to the study.

## INTERVENTIONS

The participants were exposed to four interventions for a period of 6 months. The following interventions were designed based on the intention to move HCWs from their current TTM stage to the next and to eventually move them to the action stage. To move the contemplators to pre-contemplators, the intervention aimed to improve knowledge and change the attitudes and perceptions of participants about PA. The first set of health education pamphlets and posters were distributed and maintained for a 3-month exposure time. Thereafter, a second set of posters and pamphlets were distributed, and the first of several radio shows were disseminated to help motivate and change the attitudes of this group. To move the pre-contemplators to the preparation stage, a second set of posters was released for 3 months and was aimed at motivating people to get up and kick-start their PA. The next intervention was aimed at moving preparers to the action stage. An Employee Wellness Awareness Day was organized with health check-ups, a fun run, and an aerobic dance competition to bring attention to the program and encourage participants to begin. The final intervention was to keep actors continuously moving in the maintenance stage. Radio health talks were organized weekly for 3 months to encourage, support, and answer questions about PA.

## MEASURES

The following data-collection tools were administered at baseline:

1. *Self-administered questionnaire,* which included (a) demographic characteristics (gender, age, job-category, and work experience); (b) knowledge (12 items) and attitude towards PA (25 items), and (c) TTM stages of PA.
2. *Anthropometric measures*: bodyweight (using an electronic bathroom scale) and height (using a tape measure) were measured, and body mass index (BMI) was calculated using the formula, bodyweight (kg)/height (m)^2^. BMI was classified as normal weight (18 – 24.9 kg/m^2^), overweight (25 – 29.9 kg/m^2^), and obese (≥30 kg/m^2^).
3. *Fitness testing*: a 6-min walk on a step machine and a polar heart rate monitor were used. Classifications of fitness level were based on the participants’ VO_2max._

At 6 months post-intervention, the questionnaires were repeated for sections (b) and (c) of the baseline survey plus the 39 items measuring process of change and the level of exposure to the interventions (see, read/participate in, gain information from, and use the intervention).

The questionnaires for attitudes and knowledge were tested for reliability using Cronbach’s Alpha. The results indicated that questions about both knowledge and attitudes yielded a high level of reliability (*α* = 0.81 and 0.66, respectively). The content validity of the questionnaire was also pretested among peers and non-participating staff.

The data were coded and entered into SPSS version 17.0 for analysis. Chi-square, *t* test, and logistic regressions were used to analyze the data.

## RESULTS

The demographic profile of the participants is summarized in Table 1. There were more females (81%) than males in this study. The same proportion applied to both medical and nonmedical staff. The majority of participants (66%) were older than 40 years and had work experience of greater than 10 years (66.5%). Only 26.5% of participants were classified as normal weight (BMI = 18–24.9 kg/m^2^); 26.5% were overweight (25–29.9 kg/m^2^), and nearly half of the participants (47%) were obese (≥30 kg/m^2^). There was no significant difference in the proportion of weight distribution between medical and nonmedical staff. The majority of participants (81.5%) reported a low fitness level (based on VO_2max,_ calculated according to gender and age), followed by 15.5% with a moderate fitness level and only 3% with a high physical fitness level.

**Table 1:** Sociodemographic characteristics of healthcare workers (% by column)

Variable *n* = 200		Total group (*n* = 200)	Medical staff *n* = 100	Non-medical staff *n* = 100%
		*n* (%)	%	
Gender	Males (*n* = 38)	38 (19.0)	15.0	23.0
	Females (*n* = 162)	162 (81.0)	85.0	77.0
Age	<40 (*n* = 68)	68 (34.0)	40.0	28.0
	≥40 (*n* = 132)	132 (66.0)	60.0	72.0
Work experience	<10 years (*n* = 67)	67 (33.5)	37.0	30.0
	≥10 years (*n* = 133)	133 (66.5)	63.0	70.0
BMI	Normal weight (*n* = 53)	53 (26.5)	26.0	27.0
	Overweight (*n* = 53)	53 (26.5)	30.0	23.0
	Obese (*n* = 94)	94 (47.0)	44.0	50.0
Fitness Level	Low level (*n* = 163)	163 (81.5)	73.0	90.0
	Moderate level (*n* = 31)	31 (15.5)	22.0	9.0
	High level (*n* = 6)	6 (3.0)	5.0	1.0
TTM stages	Precontemplators (*n* = 19)	19 (9.5)	12.0	7.0
	Contemplator (*n* = 94)	94 (47.0)	37.0	57.0
	Preparer (*n* = 38)	38 (19.0)	19.0	19.0
	Actor (*n* = 40)	40 (20.0)	26.0	14.0
	Maintainer (*n* = 9)	9 (4.5)	6.0	3.0

At pre-test, 9.5% of the participants were at precontemplation, 47% were at contemplation, 19% were at preparation, 20% were at action, and only 4.5% were at the maintenance stage. Medical staff (32%) reported slightly more advanced PA action stages compared with nonmedical staff (17%). When grouping the Pre-action stages (pre-contemplation, contemplation, and preparation) and the Action stages (action and maintenance), 75.5% of staff was in the Pre-action stages, and only 24.5% were in the Action stages.

### Process evaluation: exposure to interventions

One hundred sixty-three HCWs participated in the intervention phase of this study (reduced numbers were due to death, relocation, leave, and early retirement). Of the four types of interventions HCWs were exposed to, 81.0% were exposed to the posters, 74.2% to the fun-day, 72.4% to the pamphlets, and 50.3%% to the radio programs. Exposure to interventions was similar for medical and nonmedical staff, except for the fun-day, where 81.3% of the nonmedical staff was exposed compared with 67.5% of the medical staff (*p* < 0.05). When combining all interventions, the majority of both medical (90%) and nonmedical (85%) staff were highly exposed to the interventions (Table 2).

**Table 2:** The distribution of participants at post-test by the type of intervention used (% in column)

Interventions	Total (*n* = 163)	Med	Non-med	*χ* ^2^, df (*p* value)
	*n* (%)	*n* (%)	*n* (%)	
Posters	132 (81.0)	70 (84.3)	62 (77.5)	1.24, 1 (.260)
Radio	82 (50.3)	44 (53.0)	38 (47.5)	0.49, 1 (.482)
Pamphlets	118 (72.4)	63 (75.9)	55 (68.8)	1.04, 1 (.307)
Fun-day	121 (74.2)	56 (67.5)	65 (81.3)	4.04, 1 (.033)^a^

^a^Chi-squared test, significant at *p* value < 0.05

### Barriers to PA

The majority of participants (76%), irrespective of their age, gender, and job categories, reported the following barriers to exercise: lack of motivation (82.5%), lack of access to exercise equipment in the hospital environment (80%), lack of family support (57%), and lack of access to exercise equipment in the home environment (45%).

When comparing the level of exposure to interventions as the TTM stages of PA increased, the results indicated that the majority of participants who had a high exposure to the interventions (86%) progressed from their original TTM stages of PA. Similarly, 85% of nonmedical staff who were highly exposed to the intervention increased PA stages. There was no significant difference between level of exposure and PA increase for medical staff (Table 3).

**Table 3:** Exposure level to intervention and PA stage changes (% in rows)

Exposure level		PA stages increase		*χ* ^2^, df (*p* value)
		No increase	Increased	
		*n* (%)	*n* (%)	
All participants	Low exposure (*n* = 16)	7(44)	9(56)	5.61, 1 (.03)^a^
	High exposure (*n* = 143)	20(14)	123(86)	
Med	Low exposure (*n* = 8)	2(25)	6 (75)	1.40, 1 (.25)
	High exposure (*n* = 75)	8(11)	67(89)	
Non-med	Low exposure (*n* = 12)	5(42)	7(58)	3.52, 1 (.03)^a^
	High exposure (*n* = 68)	12(18)	56(82)	

^a^Chi-squared test, significant at *p* value < 0.05

In general, 38.5% of participants had excellent levels of knowledge, 15.5% had good knowledge, 34% had fair knowledge, and 12% had poor knowledge about the benefits of exercise. More medical staff had good or excellent knowledge compared with nonmedical staff (*p* < 0.05). For attitudes, 71% of HCWs had a positive attitude towards exercise, with no significant difference between medical and nonmedical staff, males and females, or young and old participants.

The comparison of the mean scores of TTM stages of PA, knowledge, and attitudes between pre-test and post-test using paired-sample *t* tests revealed that there were significant differences between pre-test and post-test scores for all three variables. The mean score of TTM stages of PA at post-test, the mean scores of knowledge, and the attitudes at post-test were significantly greater than the means at pre-test (*p* < 0.001) (Table 4).

**Table 4:** Comparison of means of PA stages, knowledge, and attitude (pre-post test)

Variables	*n*	Means	SD	Means different (pre-post)	*t* value	df	*p* value
TTM stages pre-test	163	2.64	1.03	−1.10	−18.98	162	0.000^a^
TTM stages post-test	163	3.74	1.02				
Knowledge pre-test	163	7.66	2.01	−1.64	−8.53	162	0.000^a^
Knowledge post-test	163	9.31	1.65				
Attitude pre-test	163	14.78	7.70	−3.42	−5.78	162	0.000^a^
Attitude post-test	163	18.20	5.54				

^a^Paired samples *t* test, significant at *p* value < 0.001

There was a significant change in the participants’ TTM stages of PA (*p* < 0.05) between pre- and post-interventions. Sixty-three percent (63.2%) of the participants moved to the action or maintenance stages, while few hospital staff remained in the precontemplation or contemplation stages. The proportion of nonmedical staff who increased to the action stages (24.8%) was significantly greater than the medical staff (15%). However, the medical staff in the maintenance stages increased from 3% to 32.5% over the course of the intervention, which much greater than the nonmedical staff (Table 5).

**Table 5:** Comparing medical vs. nonmedical staff: pre-test/post-test of PA stages (pre-test *n* = 200, 100 medical and 100 nonmedical staff; post-test, *n* = 163, 83 medical and 80 nonmedical staff)

Physical activity stages	Job category of hospital staff	Pre-test %	Post test %	*χ* ^2^, df (*p* value)
Pre-contemplation	Medical staff	12.0	6.0	38.88, 1 (.000) ^a^
	Nonmedical staff	7.0	1.3	
Contemplation	Medical staff	37.0	6.0	8.19, 1 (.004)^b^
	Nonmedical staff	57.0	13.8	
Preparation	Medical staff	19.0	16.9	3.02, 1 (.07)
	Nonmedical staff	19.0	30.0	
Action	Medical staff	26.0	41.0	24.07, 1 (.000) ^a^
	Nonmedical staff	14.0	38.8	
Maintenance	Medical staff	6.0	32.5	19.66, 1 (.000) ^a^
	Nonmedical staff	3.0	16.2	

^a^Chi-squared test, significant at *p* value < 0.001

^b^Chi-squared test, significant at *p* value < 0.01

The potential associations between selected variables and the association with increases in PA stages were assessed. Only three variables were found to be significantly associated with increased levels of PA stages: feelings about weight, PA status at baseline, and exposure to the interventions. Of those participants whose PA increased, 41.2% felt bad about their weight, 78.2% were in the pre-action stages, and 90.4% had high levels of exposure to the interventions (Table 6).

**Table 6:** The association between selected variables and the increased (move up) TTM stages of physical activity among hospital staff (percentage by column)

	No increase PA stages %	Increase PA stages %	*χ* ^2^, df (*p* value)
Age: young 18–39 years (*n* = 49)	25.9	30.9	0.26, 1 (.608)
Work experiences <10 years (*n* = 46)	29.6	27.9	0.03, 1 (.859)
Female (*n* = 133)	88.9	80.1	1.45, 1 (.284)
Medical staff (*n* = 83)	37.0	53.7	2.50, 1 (.114)
Work day shift (*n* = 148)	88.9	91.2	0.14, 1 (.706)
Healthy: self-reported (*n* = 121)	19.0	81.0	2.03, 1 (.154)
Non-smoker (*n* = 145)	92.6	88.2	0.43, 1 (.509)
Alcohol drinker (*n* = 116)	70.4	71.3	0.01, 1 (.920)
BMI: overweight and obese (*n* = 119)	77.8	72.1	0.37, 1 (.541)
Feel bad about their weight (*n* = 73)	63.0	41.2	4.32, 1 (.034)^a^
Tried to lose weight (*n* = 70)	40.7	43.4	0.06, 1 (.800)
PA in Pre-action at baseline (*n* = 124)	21.8	78.2	10.18, 1 (.001)^b^
High exposure to intervention (*n* = 143)	74.1	90.4	5.61, 1 (.001) ^b^

^a^Chi-squared test, significant at *p* value < 0.05

^b^Chi-squared test, significant at *p* value < 0.01

The three variables found to have significant correlations with increased PA stages were selected for the prediction model for increasing PA level using multivariable logistic regression analysis. The model initially estimated an 83.4% prediction value. When entering three variables (exposure to intervention, feeling bad about weight, and TTM stage at baseline), the Omnibus test of model coefficients was significant (*χ* ^2^ = 24.95, df = 3, *p* value < 0.001) with an *R* ^2^ of 0.24. The prediction value increased to 86.5%. Participants who reported high exposure to the interventions experienced four times greater increase in PA stages than those who had low exposure to the interventions, with an OR (Exp (B)) of 4.14, 95% CI = 1.31–13.41 (*p* value < 0.05) (Table 7).

**Table 7:** Model predicting increasing level of physical activity (PA stages)

Variables in equation	*B*	SE	Wald	df	Sig.	Exp (B)	95% C.I. for EXP(B)	
							Lower	Upper
Pre-action stage at baseline	−19.82	6,302.53	0.00	1	0.997	0.000	0.000	0.000
Feel bad about their weight	−0.817	0.463	3.12	1	0.077	0.442	0.178	1.094
High exposure to intervention	1.423	0.588	5.85	1	0.016*	4.151	1.311	13.144
Constant	20.34	6,302.53	0.00	1	0.997	6.813E		

Variable(s) entered on step 1: TTM, feel bad about weight, exposed to intervention

*p value < 0.05, level of significance

Binary logistic regression was used to determine the predictive validity of TTM at pre- and post-tests. The overall accuracies of TTM at pre-test were classified correctly by the TTM at post-test with an average of 66.9% for all participants (*p* < 0.05). The results also indicated a difference in the accuracy between medical and nonmedical staff (78.3% and 65%, respectively, *p* < 0.05) (Table 8).

**Table 8:** The predictive validity of using TTM in PA stage changes

Group	Observed TTM at post-test	Predicted PA at post-test		% Correct	*B* (Wald)	Sig.	Exp (B)	95% CI
		Pre-action^a^	Action^b^					
All staff	Pre-action	47	11	81.0	1.44 (27.37)	0.000	4.01	2.45–7.21
	Action	43	62	59.0				
	Overall % predicted correctly			66.9				
Medical staff	Pre-action	6	16	27.3	1.36 (13.86)	0.000	3.90	1.90–7.98
	Action	2	59	96.7				
	Overall % predicted correctly			78.3				
Non-medical staff	Pre-action	30	6	83.3	1.48 (12.23)	0.000	4.41	1.92–10.15
	Action	22	22	50.0				
	Overall % predicted correctly			65.0				

^a^Pre-action = pre-contemplation + contemplation + preparation

^b^Action = action + maintenance

## DISCUSSION

The aim of this study was to determine the predictive validity and effects of using TTM to increase PA among HCWs in South Africa. This study highlights the serious prevalence of overweight and obesity among HCWs (73.5%), with women being more obese than men irrespective of age, and no significant difference in weight distribution between medical and nonmedical staff. The majority of HCWs were physically inactive at baseline, which may be contributing to the high prevalence of obesity in this group.

HCWs are regarded as role models by their communities and are expected to maintain a healthy lifestyle. Sadly, that is not the case in South Africa, as they are also included in the countrywide obesity problem. Poor knowledge about the benefits of increasing one’s level of PA has been suggested to contribute to low levels of PA by many researchers; interestingly, the results of this study showed no significant difference in the levels of PA between medical staff (with excellent knowledge) and nonmedical staff (with poor knowledge). There is a need for the development of cost-effective interventions to promote a healthy lifestyle, which includes good nutrition and increased levels of PA. Theory-based interventions have been successful in changing behavior throughout the world; it is therefore important for the South African healthcare system to implement successful interventions that are theory-based to effect behavior change. This study therefore aimed to demonstrate the validity of using one of the effective theories (TTM) as a guideline for increasing the level of PA among HCWs in the South African context. TTM was used to identify the stages of readiness of HCWs at pretest and to create stage-specific interventions to increase the HCWs’ level of PA.

HCWs in this study reported that the hospital was not a conducive environment for exercising. Although there is a movement by the South African government to improve employee wellness, there is still a great challenge in enforcing this change. Hospitals should be seen as health-promoting institutions that not only look after patients but also encourage staff to exercise by creating space and allowing time off for staff to participate in wellness programs, especially those aimed at increasing the level of PA. This view is in line with a previous study, which reports that a worksite PA program with an individual approach may increase PA levels of workers [15]. Two other recent studies also advocated that worksites should engage in increasing environmentally focused PA promotion activities, such as education, and structural integration of PA into workplace routines [16, 17]. Titze, Martin, Seiler, and Marti [18] conducted a study on improving PA at a worksite and found a significant 21% improvement in the staff members’ baseline PA stages to more active stages. They also concluded that there should be a diversified intervention program in a worksite setting that encourages staff to become more physically active during work and leisure time [18].

In the present study, the level of exposure to interventions was high, with the majority of staff being exposed to three to four interventions, resulting in significant changes in their behavior (*p* < 0.05) post-intervention. Web et al. found that interventions that incorporated more behavior change techniques had greater effects compared with interventions that incorporated fewer techniques (*p* < 0.001) [19]. Of the four interventions designed to change behavior of participants in this study, posters and pamphlets were the most used. Both interventions contained creative pictures with captions, which drew a lot of attention and interest from participants to ensure that they were not bored or lost interest.

Post-intervention, there was a significant improvement in the exercise behavior of HCWs, and most staff progressed from the Pre-action to the Action stage. Similarly, Woods, Mutrie, and Scott [20] and other researchers [21, 22] have found that participants were able to move from their initial stage following TTM-based interventions. These results indicate that TTM-based interventions can be used successfully to change the PA behavior of HCWs in South Africa, despite their diverse cultural backgrounds. There is an assumption that an individual will not change his/her behavior unless he/she perceives positive effects from the behavioral change that outweigh the negative effects [20]. The results of some cross-sectional studies generally indicated that pros increase and cons decrease from pre-contemplation to maintenance [12, 23, 24].

The results of this study revealed that the overall accuracies of PA stages at pre-test were classified correctly by PA stages at post-test by an average of 66.9%. Towers et al. [12] conducted two studies predicting the stages of change for men and women and found similar results; the results for action/maintenance revealed an overall model of one factor (affect temptation) associated with the stage of change that was statistically reliable in distinguishing between the action and maintenance stages [12]. The model correctly classified 73.5% of the cases for men and 62.5% cases for women. The results for maintenance/termination revealed the overall model of three predictors (barriers to efficacy, experiential processes of change, and affect temptation) that were statistically reliable in distinguishing between maintenance and termination for men, whereas the results for women indicated that experiential processes of change was the only predictor that was statistically reliable in distinguishing between action and maintenance [12].

## CONCLUSION

More attention should be directed to the health of HCWs in South African healthcare institutions. The use of TTM as a guide for implementing tailored PA interventions was demonstrated to be useful in progressing HCWs from their original TTM stages of PA in this study. Hospitals must be seen as promoting healthy lifestyles by creating better environments that are conducive to exercise. The level of exposure, knowledge, and attitudes were constructs that correctly predicted an improvement in TTM stages post-intervention.
